# Zebrafish model of *KRAS*-initiated pancreatic cancer

**DOI:** 10.1080/19768354.2018.1530301

**Published:** 2018-10-08

**Authors:** Joon Tae Park, Steven D. Leach

**Affiliations:** a Division of Life Sciences, College of Life Sciences and Bioengineering, Incheon National University, Incheon, Korea; b Department of Molecular and Systems Biology, Norris Cotton Cancer Center, Geisel School of Medicine at Dartmouth, Hanover, NH, USA

**Keywords:** Pancreatic cancer, PanIN, zebrafish, KRAS

## Abstract

Pancreatic cancer constitutes a genetic disease in which somatic mutations in the *KRAS* proto-oncogene are detected in a majority of tumors. *KRAS* mutations represent an early event during pancreatic tumorigenesis that crucial for cancer initiation and progression. Here, we established a zebrafish pancreatic cancer model that highly recapitulates human pancreatic intraepithelial neoplasia (PanIN) development. We established a novel system combining CRE/Lox technology with the GAL4/UAS system to express oncogenic KRAS in the *ptf1a* domain temporarily. In this system, zebrafish developed PanIN at an 11.1% rate by 24 and 36 weeks after KRAS^G12V^ induction. The histological and immunohistochemical profiles of these experimental tumors bore striking resemblance to human PanIN. Within the whole abnormal area, the entire spectrum of differentiation ranging from PanIN-1 to PanIN-3 was noted. Immunohistochemical analysis including Alcian blue, CK-18, cadhedrin-1, and DCLK1 staining confirmed the PanIN region as a characteristic pancreatic cancer precursor lesion. Taken together, these findings demonstrate that this zebrafish model may offer the possibility of an experimental and preclinical system to evaluate different strategies for targeting pancreatic tumors and finally improve the outcome for the patients with pancreatic tumors.

## Introduction

Pancreatic cancer is the fourth leading cause of cancer death in the United States, with a very low rate of survival following diagnosis (Jemal et al. 2008). Most pancreatic cancers comprise infiltrating pancreatic ductal adenocarcinomas (PDAC), which is considered to arise from ductal precursor lesions termed pancreatic intraepithelial neoplasia (PanIN) (Hruban et al. 2005). In humans, 45% of early PanIN lesions and 90% of infiltrating PDAC harbor oncogenic *KRAS* mutations (Hruban et al. 1993; Hruban et al. 2000; Jones et al. 2008). Several mouse models for *KRAS*-mediated pancreatic cancer have been generated. A major breakthrough emerged from an approach targeting an oncogenic *KRAS* in presumed progenitor cells, using *pdx1* or *ptf1a* drivers (Hingorani et al. 2003). These mutant mice developed PanIN lesions similar to those seen in humans, underscoring the fact that oncogenic *KRAS* functions as a critical initiator of pancreatic tumorigenesis (Hingorani et al. 2003). The dramatic progress of mouse models for pancreatic cancer arouses the expectation that these models could be used to develop a new drug against PDAC, as preclinical trials of anticancer drugs in animal models constitute an important step in the drug development (Aguirre et al. 2003; Hingorani et al. 2003; Hruban et al. 2006). However, the findings of most preclinical trials of the new drugs that showed a good efficacy against PDAC in a mouse model were not confirmed during the clinical phase (Kapischke and Pries 2008). Addressing this fundamental problem will likely require the development of a new platform that could be highly utilized in drug development.

The zebrafish has emerged as an excellent model organism for the study of human cancer. Zebrafish show the same characteristics as human cancers, such as genomic instability, invasiveness, and metastasis (Le et al. 2007). Moreover, the optical transparency of the zebrafish embryo makes it possible to track the locations or activities of genes of interest (Beis and Stainier 2006; Liu et al. 2008). For example, tagging the transgene with a fluorescent protein allows for the real-time observation of tumor progression and invasion. It also makes it possible to monitor drug efficacy *in vivo* and to assess the severity of a specific phenotype visually. In our previous studies, we established two *KRAS*-initiated pancreatic cancer models in zebrafish (Park et al. 2008; Liu and Leach 2011). Our first model used a genomic bacterial artificial chromosome (BAC) (CH211-142H2) spanning the zebrafish *ptf1a* locus to express the human KRAS^G12V^ mutant in the exocrine pancreas (Park et al. 2008). After oncogenic *KRAS* was expressed in developing zebrafish pancreas, pancreatic progenitor cells failed to undergo normal exocrine differentiation, leading to the subsequent formation of invasive pancreatic cancer. However, this model induced predominant acinar cell carcinomas as opposed to the classical pancreatic cancer development (Park et al. 2008). Our second model used the GAL4/UAS system to express the human KRAS^G12V^ mutant in the exocrine pancreas (Liu and Leach 2011). Instead of expressing the human KRAS^G12V^ mutant directly from *ptf1a* regulatory elements, we used a novel GAL4/UAS approach that allows the simultaneous expression of human KRAS^G12V^ mutant in *ptf1a*-expressing pancreatic cell types. However, this model also induced predominant acinar cell carcinomas with a few tumors with mixed acinar and ductal differentiation (Liu and Leach 2011).

In the present study, we combined CRE/Lox and GAL4/UAS systems to establish the first *KRAS*-initiated pancreatic cancer model in zebrafish that highly recapitulates human PanIN development. As this model showed the whole spectrum of PanINs, it holds promise as an experimental model system to evaluate different strategies for targeting pancreatic cancers.

## Materials and methods

### Generation of transgenic zebrafish

All experiments involving zebrafish were approved by the Johns Hopkins University Institutional Animal Care and Use Committee. Fish were raised and maintained under standard laboratory conditions. The following strains were established and/or utilized: *Tg* (*ptf1a:CRE^ERT2^*; cryaa:Venus) (herein *ptf1a:CRE^ERT2^*), *Tg (ubb:lox-Nuc-eCFP-stop-lox-GAL4-VP16)* (herein *LSL-GAL4*), and *Tg* (*UAS:eGFP-KRAS^G12V^*) (herein *UAS-KRAS^G12V^*) (Liu and Leach 2011). Larvae were anesthetized in 0.16% tricaine (3-aminobenzoic acid ethylester, A-5040, Sigma, USA). Adult zebrafish were euthanized by induction of tricaine anesthesia followed by placement in an ice bath, consistent with recommendations of the Panel on Euthanasia of the American Veterinary Association.

### Analysis of tumor in adult fish

Transgenic adult male *Tg* (*LSL-GAL4)* fish were outcrossed to a transgenic adult female fish *Tg* (*UAS-KRAS^G12V^*) line. The double transgenic adult *Tg* (*LSL-GAL4; UAS-KRAS^G12V^)* females were outcrossed to transgenic adult *Tg* (*ptf1a:CRE^ERT2^*) male*.* Progeny of this cross, *Tg (ptf1a:CRE^ERT2^; LSL-GAL4; UAS-KRAS^G12V^),* were treated with 5 µM 4-hydroxytamoxifen (4-OHT, T176, Sigma) in E3 medium two times between 21 and 28 days post-fertilization (dpf). A random subset of fish was anesthetized and sacrificed at 12-, 24-, and 36-week time points after 4-OHT treatment for histologic evaluation. PanIN regions were quantitated using ImageJ (Collins 2007).

### Immunohistochemistry and immunofluorescence

Immunohistochemistry and immunofluorescence analyses were performed on 5 μm paraffin-embedded sections as described previously (Lin et al. 2004). Alcian blue staining was performed according to the manufacturer’s instructions (Sigma). Primary antibodies used for immunohistochemistry were rabbit anti-Keratin18 (Anaspec Inc., USA; 55357, 1:200) and rabbit anti-phospho AKT (Cell Signaling Technology, USA, 4060S, 1:400). The secondary antibody was biotin-conjugated anti-rabbit (Jackson Immunoresearch Laboratories, USA; 711-066-152, 1:500). For the ABC reaction, ABC kit Vectastain PK-6100 from Vector Labs (USA) was used. Primary antibodies used for immunofluorescence were rabbit anti-DCLK1 (Abcam, USA; ab37994, 1:200), rabbit anti-cadhedrin1 (Anaspec Inc.; 55615, 1:200) and rabbit anti-PCNA (Santa Cruz Biotechnology, USA; sc-7907, 1:500). Secondary antibodies used for immunofluorescence were Cy5-conjugated anti-rabbit antibodies (Jackson Immunoresearch Laboratories; 711-175-152, 1:400) and Cy3-conjugated anti-mouse antibodies (Jackson Immunoresearch Laboratories; 715-165-150, 1:400).

## Results

### Identification of the PanIN regions in *Tg (ptf1a:CRE^ERT2^; LSL-GAL4; UAS-KRAS^G12V^)* fish

**Figure 1.**
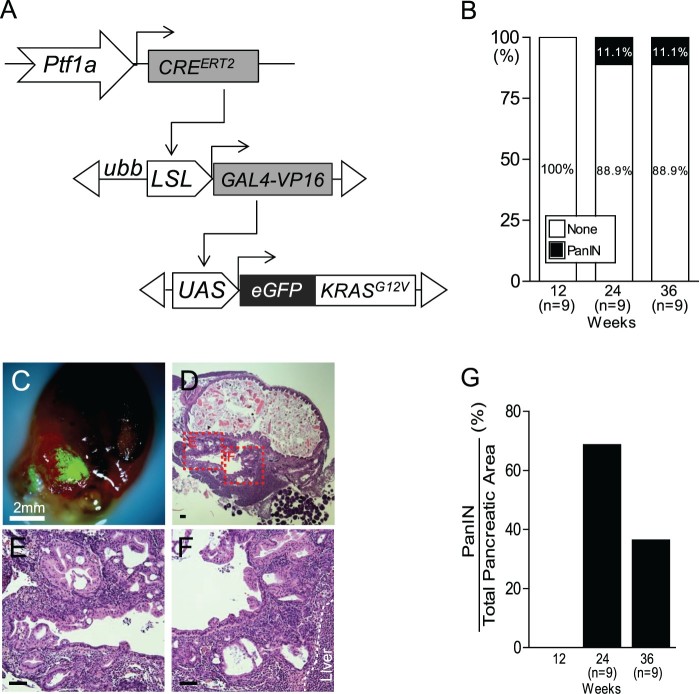
Identification of tge PanIN region in *Tg* (*ptf1a:CRE^ERT2^; LSL-GAL4; UAS-KRAS^G12V^*) fish. (A) Schematic of the *ptf1a* CRE-driver line,*Tg* (*ptf1a:CRE^ERT2^*), the CRE-responder line *Tg* (*LSL-GAL4*), and the GAL4-responder line *Tg* (*UAS-KRAS^G12V^*). (B) Quantiﬁcation of PanIN induction frequency in *Tg* (*ptf1a:CRE^ERT2^; LSL-GAL4; UAS-KRAS^G12V^*) fish. (C) Dissected abdominal viscera with an eGFP-positive tumor from KRAS^G12V^. Scale bars: 2 mm. (D–F) The histological profiles of tumors bear striking resemblance to human PanIN. Boxed areas indicate regions depicted at higher magnification in adjacent images. Scale bars: 50 μm. (G) Quantiﬁcation of PanIN region vs. total pancreatic area in *Tg* (*ptf1a:CRE^ERT2^; LSL-GAL4; UAS-KRAS^G12V^*) fish.

To control KRAS^G12V^ expression in a tissue-specific manner, we generated a conditional GAL4-VP16 transactivator under the control of the *ubiquitin b (ubb)* promoter with a lox-stop-lox (LSL) cassette inserted between the promoter and the GAL4-VP16*, Tg (ubb:LSL:GAL4-VP16)* (herein *LSL-GAL4*). Next, we crossed the *Tg (LSL-GAL4)* line with the *Tg (UAS:eGFP-KRAS^G12V^)* (herein *UAS-KRAS^G12V^*) line to direct eGFP-KRAS^G12V^ expression (Liu et al. 2011). To activate the *KRAS* gene in zebrafish pancreatic progenitor cells, we used the inducible *Tg (ptf1a:CRE^ERT2^)* line, which induces the expression of the transgene in *ptf1a*-expressing cells of the zebrafish pancreas after 4-OHT treatment (Wang et al. 2015). Finally, we established a triple transgenic fish *Tg (ptf1a:CRE^ERT2^: LSL-GAL4: UAS-KRAS^G12V^).* This novel activation system induced the expression of GAL4 under the control of the *ubb* promoter after excision of an LSL cassette by CRE in the *ptf1a* domain (Figure 1(A)). In turn, GAL4 proteins bind to their specific recognition sequence, upstream activating sequence (UAS), and stimulate transcription of oncogenic KRAS^G12V^ (Figure 1(A)).

To induce the expression of eGFP-KRAS^G12V^, triple transgenic fish *Tg (ptf1a:CRE^ERT2^; LSL-GAL4: UAS-KRAS^G12V^)* were treated with 5 µM 4-OHT in the E3 medium. A random subset of fish was anesthetized periodically and sacrificed at 12-, 24-, and 36-week time points after 4-OHT treatment. At 12 weeks after 4-OHT treatment, all examined fish showed histologically normal pancreas and no evidence of tumor formation in any organ (*n* = 9, data not shown). At 24 and 36 weeks after 4-OHT treatment, 1/9 fish (11.1%) developed PanIN, which recapitulated human PanIN (Figure 1(B)). eGFP fluorescence in the pancreas was observed from the dissected abdominal viscera of zebrafish that developed PanIN. This fluorescence was sufficiently strong to be distinguishable from autofluorescence in the intestinal tube or spleen under a fluorescence dissecting microscope (Figure 1(C)). In PanIN region, the full spectrum of PanIN was observed in 68.7% and 36.4% of the total pancreatic area at 24 and 36 weeks after 4-OHT treatment, respectively (Figure 1(D–G)). Taken together, these data substantiate KRAS^G12V^ mutation as an initiating event in the PanIN/PDAC sequence in pancreatic cancer.

### Histological and immunohistochemical profiles of PanIN regions

**Figure 2.**
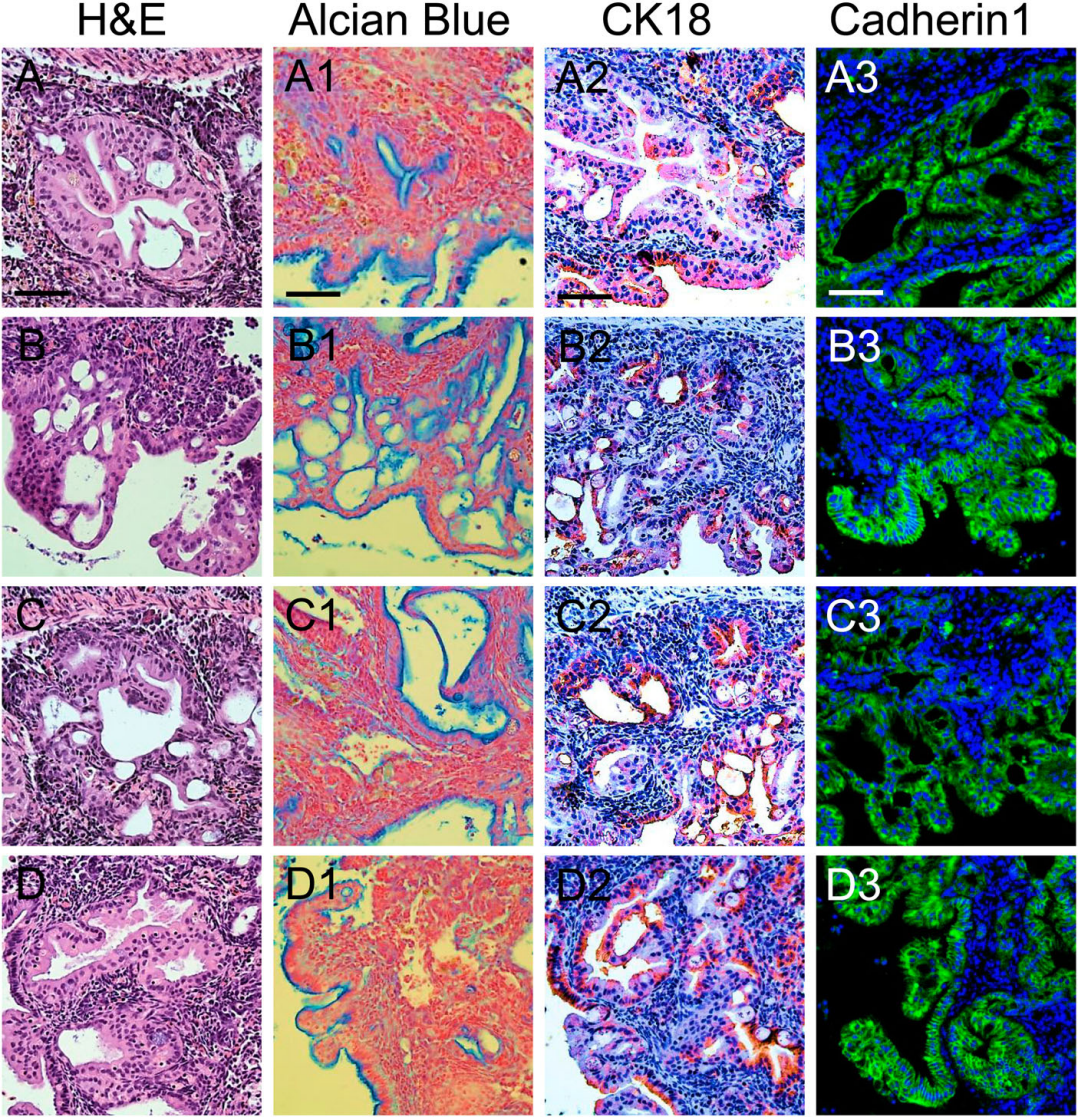
Histological and immunohistochemical profiles in PanIN regions. (A–D) Several grades of differentiation ranging from PanIN-1 to PanIN-3 in PanIN regions, as indicated by hematoxylin and eosin (H&E) staining. (A1–D1) Alcian blue staining. (A2–D2) CK-18 staining. (A3–D3) Cadherin1 staining

To determine whether these experimental tumors resembled human PanIN, their histological and immunohistochemical profiles were examined. In the whole abnormal area, several grades of differentiation ranging from PanIN-1 to PanIN-3 were noted (Figure 2(A–D)). In PanIN-3 lesions, significant nuclear atypia and complete loss of polarity were observed, such that true cribriforming; i.e. budding off small clusters of epithelial cells into the lumen, obscured the distinction between luminal and basal boundaries (Figure 2(A,B)). The development of papillary, micropapillary or pseudostratified ductal lesions without nuclear atypia identified these as PanIN-1B (Figure 2(C,D)).

To identify the characteristics of PanIN regions, Alcian blue staining, immunohistochemistry against CK18, and immunofluorescence against cadherin1 were performed. Consistent with human PanIN, an abundant mucin content of PanIN was demonstrated by intense Alcian blue staining as a marker of mucin accumulation (Figure 2(A1–D1)). PanIN regions also showed highly positive staining with ductal specific marker, CK-18, suggesting their characteristics as ductal cells (Figure 2(A2–D2)). Furthermore, intense cadherin1 staining was observed in the membrane of PanIN regions, confirming their identity as a characteristic pancreatic cancer precursor lesions (Figure 2(A3–D3)).

**Figure 3.**
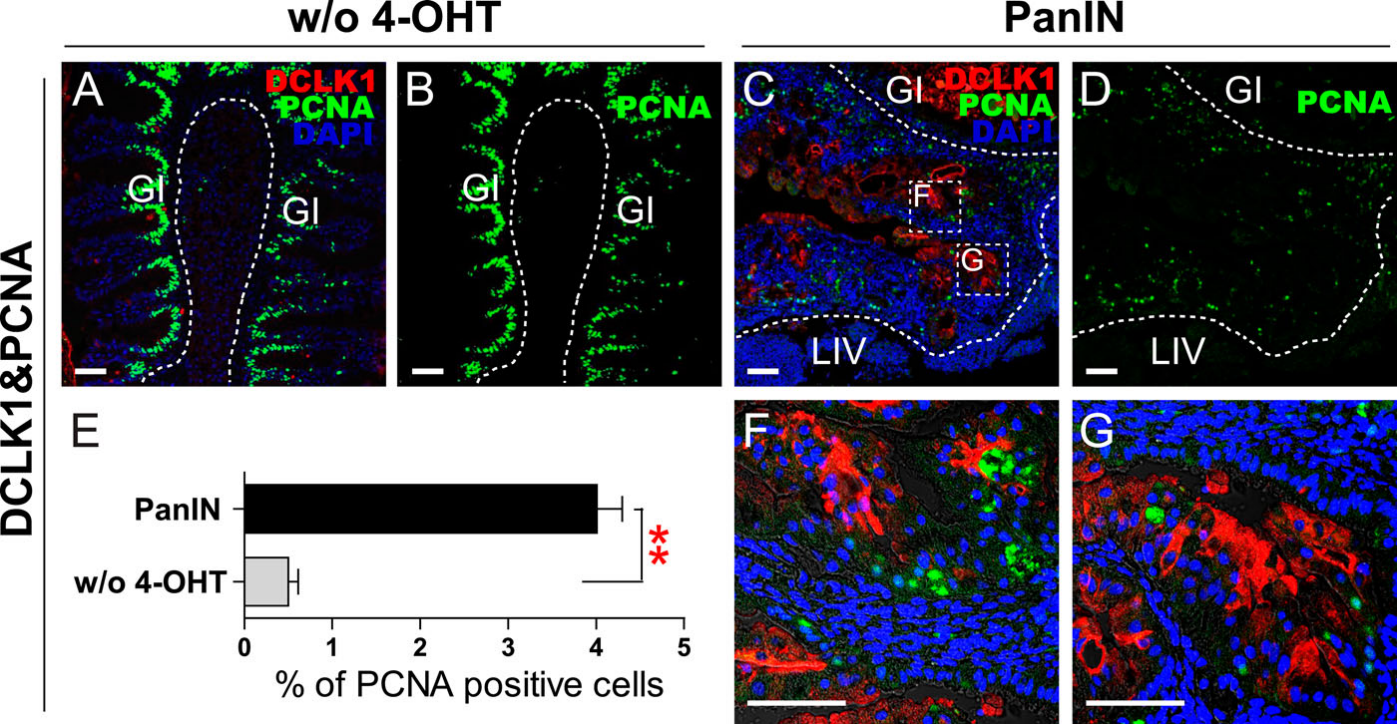
DCLK1 and PCNA staining in PanIN regions. (A and B) DCLK1 and PCNA staining in groups without 4-OHT treatment (w/o 4-OHT) as controls. Scale bars: 50 μm. (C and D) DCLK1 and PCNA staining in PanIN regions. Boxed areas indicate regions depicted at higher magnification in adjacent images. Scale bars: 50 μm. (E) Quantiﬁcation of PCNA positive cells in groups without 4-OHT treatment, and in PanIN regions. (**P < .01, one-way ANOVA). (F and G) Magnified region of boxed areas in Figure 3(C). Scale bars: 50 μm.

DCLK1, a marker for gastric tuft cells, is also observed in the surface epithelium of PanIN lesions and in the intervening stroma in human pancreatic adenocarcinoma (Sureban et al. 2011). DCLK1 staining was performed on the PanIN in addition to pancreas region from groups without 4-OHT treatment as a control. In the control group, staining was observed in tuft cells of the GI tract but not in the pancreas (Figure 3(A)). However, in the PanIN and the surrounding abnormal regions, DCLK1 staining was observed (Figure 3(C,F,G)). The proliferative index of PanIN was assessed by expression of PCNA. In the group without 4-OHT treatment, the gastrointestinal epithelium underwent rapid cell turnover and the intestinal stem cells situated in the crypt of the fingerlike intestinal villi showed highly positive PCNA staining (Figure 3(A,B)). However, the percentage of PCNA positive cells in the pancreas was low, measuring 0.50% (Figure 3(E)). Conversely, in PanIN and surrounding abnormal regions, the basal level of PCNA expression was high, measuring 4.01% (Figure 3(C–E)).

**Figure 4.**
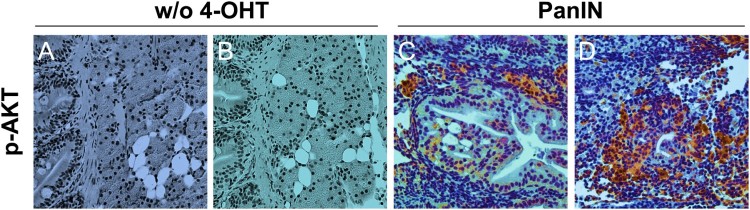
Characterization of the *KRAS* downstream signaling pathway in PanIN regions. (A and B) Phospho-AKT staining in groups without 4-OHT treatment (w/o 4-OHT) as controls. Scale bars: 50 μm. (C and D) Phospho-AKT staining in PanIN regions. Scale bars: 50 μm.

In addition, to determine the status of downstream signaling pathways known to be activated by oncogenic *KRAS*, we assessed levels of phospho-AKT using immunohistochemistry. In contrast to the infrequent AKT phosphorylation observed in groups without 4-OHT treatment (Figure 4(A,B)), PanIN regions showed widespread labeling for phospho-AKT (Figure 4(C,D)).

## Discussion

In this study, we established the first *KRAS*-initiated pancreatic cancer model that closely recapitulates human PanIN. To reach the breakthrough in establishing zebrafish PanIN/PDAC model, we used a combination of CRE/Lox and GAL4/UAS systems. CRE/Lox system temporarily activated the GAL4 transactivator in the pancreatic progenitor region, whereas the GAL4/UAS system amplified transcription of the target gene. Thus, oncogenic KRAS^G12V^ expression could be enhanced within the presumed *ptf1a* region of the pancreas. This strategy induced a *KRAS*-initiated pancreatic cancer model in zebrafish as evidenced by the development of the whole spectrum of PanIN. The histological and immunohistochemical profiles of these PanIN bore striking resemblance to human PanIN. Thus, we propose that the established *KRAS*-initiated pancreatic cancer model, generated by use of the combination of CRE/Lox and GAL4/UAS systems, now provides a preclinical platform to test various strategies for pancreatic cancer and finally improve outcomes for pancreatic cancer patients.

However, we also admit that our established *KRAS*-mediated model also needs some modifications, as this combined system induced PanIN at relatively a low rate (11.1%). As the lower incidence may impede its usage for drug screening and development, we propose several approaches to increase the induction frequency in our zebrafish model. First, a *p53* mutant background may increase the PanIN incidence in the zebrafish model. *p53* mutation occurs in approximately 50–75% of human PDAC and plays a critical role in cell cycle regulation by inhibiting growth arrest (Ryan et al. 2001). The significance of p53 mutation in pancreatic cancer is highlighted by the finding that the endogenous expression of both oncogenic *KRAS* and *p53* mutant in the mouse pancreas dramatically shortened median survival months as compared with *KRAS* activation alone (Hingorani et al. 2005). Second, the coactivation of *Notch* and *KRAS* may increase the frequency of PanIN formation in the zebrafish model. *Notch* activation is known to confer sensitivity to oncogenic KRAS by inducing the phenotypic plasticity of adult exocrine cells (Miyamoto et al. 2003; Siveke et al. 2008). Support for this finding is evident from the observation that coactivation of *Notch* and oncogenic *KRAS* dramatically increased PanIN formation compared with *KRAS* activation alone in a mouse model (De La O et al. 2008). Third, cerulean treatment may increase the frequency of PanIN in the zebrafish model, as ceruline-induced acute or chronic pancreatitis accelerated oncogenic *KRAS*-induced PanIN/PDAC formation (Carriere et al. 2007; Guerra et al. 2007; Friedlander et al. 2009; Morris et al. 2010). Furthermore, concurrent with animal model data, patients with pancreatitis show a higher risk of developing PanIN/PDAC (Raimondi et al. 2010). Taken together, with the proposed approaches, the induction frequency of PanIN formation is warranted to be increased to be used as a platform for the functional annotation of somatic mutations identified in pancreatic cancer genomes.

The zebrafish has emerged as an excellent model organism in the study of cancer biology over the last several decades. Traditionally, the focus of zebrafish research was on developmental biology because of the clear advantages afforded by this organism, such as the large brood size, transparent embryos, *ex utero* development of the embryo, and short life cycle. The long history of research in the developmental biology of the zebrafish has led to the identification of diseases similar to those in humans, especially cancer, with a closely histopathological and molecular similarity being observed between human and zebrafish tumors (Amatruda et al. 2002). Furthermore, zebrafish models are considered to be well suited for preclinical high-throughput drug screening, as the optical transparency of the zebrafish embryo makes it possible to monitor the effect of the drug on tumors with regard to their initiation, progression, and metastasis (Beis and Stainier 2006; Liu et al. 2008). Until recently, the preferred drugs for use against pancreatic cancer have been capecitabine and oxaliplatin (Bullock et al. 2017). However, the combination of chemotherapy with capecitabine and oxaliplatin induces duodenal ulcer bleeding and deterioration of general medical condition including anorexia and neutropenia (Chung et al. 2017). Thus, there is a need for better drugs that can be used alone or in combination with capetiabine/oxaliplatin. We propose that our *KRAS-*induced-pancreatic model may serve as a useful tool for preclinical high-throughput drug screening against pancreatic cancer. In particular, our model affords the ability to monitor the severity of dedifferentiation or transdifferentiation in oncogenic *KRAS-*induced-pancreatic progenitor cells, providing an avenue for *in vivo* testing of drug efficacy.

In summary, we successfully developed a novel system combining CRE/Lox technology with the GAL4/UAS system to establish an oncogenic *KRAS*-initiated pancreatic cancer model. Our novel system demonstrated that *KRAS^G12V^*-responsive pancreatic progenitor cells could induce PanIN, constituting a precursor region of PDAC. This zebrafish cancer model thus provides an experimental and preclinical model system to investigate the basic biology of pancreatic cancer and identify potential therapeutic targets.

## Author contributions

JTP and SDL conceived of and designed the experiments. JTP performed the experiments. JTP and SDL wrote and edited the paper.
